# Immune Dysregulation as a Cause of Autoinflammation in Fragile X Premutation Carriers: Link between *FMRI* CGG Repeat Number and Decreased Cytokine Responses

**DOI:** 10.1371/journal.pone.0094475

**Published:** 2014-04-09

**Authors:** Milo Careaga, Destanie Rose, Flora Tassone, Robert F. Berman, Randi Hagerman, Paul Ashwood

**Affiliations:** 1 Department of Medical Microbiology and Immunology, University of California Davis, Davis, California, United States of America; 2 The M.I.N.D. Institute, University of California Davis, Davis, California, United States of America; 3 Department of Biochemistry and Molecular Medicine, University of California Davis, Davis, California, United States of America; 4 Department of Neurological Surgery, University of California Davis, Davis, California, United States of America; 5 Department of Pediatrics University of California Davis, Davis, California, United States of America; French National Centre for Scientific Research, France

## Abstract

**Background:**

Increased rates of autoinflammatory and autoimmune disorders have been observed in female premutation carriers of CGG repeat expansion alleles of between 55–200 repeats in the fragile X mental retardation 1 (*FMR1*) gene. To determine whether an abnormal immune profile was present at a cellular level that may predispose female carriers to autoinflammatory conditions, we investigated dynamic cytokine production following stimulation of blood cells. In addition, splenocyte responses were examined in an *FMR1* CGG knock-in mouse model of the fragile X premutation.

**Methods:**

Human monocyte and peripheral blood leukocytes (PBLs) were isolated from the blood of 36 female *FMR1* premutation carriers and 15 age-matched controls. Cells were cultured with media alone, LPS or PHA. In the animal model, splenocytes were isolated from 32 CGG knock-in mice and 32 wild type littermates. Splenocytes were cultured with media alone or LPS or PMA/Ionomycin. Concentrations of cytokines (GM-CSF, IL-1β, IL-6, IL-10, IL-13, IL-17, IFNγ, TNFα, and MCP-1) were determined from the supernatants of cellular cultures via Luminex multiplex assay. Additionally, phenotypic cellular markers were assessed on cells isolated from human subjects via flow cytometry.

**Results:**

We found decreases in cytokine production in human premutation carriers as well as in the *FMR1* knock-in mice when compared with controls. Levels of cytokines were found to be associated with CGG repeat length in both human and mouse. Furthermore, T cells from human premutation carriers showed decreases in cell surface markers of activation when compared with controls.

**Conclusions:**

In this study, *FMR1* CGG repeat expansions are associated with decreased immune responses and immune dysregulation in both humans and mice. Deficits in immune responses in female premutation carriers may lead to increased susceptibility to autoimmunity and further research is warranted to determine the link between *FMR1* CGG repeat lengths and onset of autoinflammatory conditions.

## Introduction

The fragile X mental retardation 1 (*FMR1*) gene, encoding for the *FMR1* protein (FMRP), contains a trinucleotide (CGG) repeat element in the 5′ untranslated region of *FMR1.* The length of the CGG repeat in most individuals is less than 55 repeats. However, carriers of a full mutation have CGG repeat expansions greater than 200, resulting in methylation and silencing of the gene, an absence of FMRP, and the subsequent development of fragile X syndrome (FXS). In between are premutation carriers who have between 55–200 CGG repeats on *FMR1*, increased *FMR1* mRNA expression levels, slightly reduced levels of FMRP, and are at risk for developing a number of neurological and physiological symptoms [1]. The prevalence of premutation alleles in the general population is surprisingly high, with rates in men estimated to be as 1 in 250 and in women as high as 1 in 130 [2].

The cause of the premutation phenotypes have been attributed to elevated levels of *FMR1* mRNA leading to sequestration of proteins including Drosha and DGCR8, critical for processing of miRNAs [3]. For example, sequestration of Drosha and DGCR8 in brain tissue from premutation carriers is associated with decreased miRNA levels in FXTAS [3]. A recent report provides evidence for an additional mechanism of toxicity resulting from non-AUG-initiated (RAN) translation of CGG repeat expansions leading to production of a toxic polyglycine peptide [4]. There is also evidence that psychiatric problems including anxiety and ADHD may be relate to lower FMRP levels, particularly those with a higher CGG repeat [5].

A number of phenotypes are associated with the expanded CGG allele. One of the earliest associated disorders observed in premutation carriers was primary ovarian insufficiency (FXPOI), noted in approximately 16–20% of female carriers [6]–[9]. Fragile X-associated tremor/ataxia syndrome (FXTAS) was described later in premutation carriers who developed intention tremor and gait ataxia [10], [11]. FXTAS occurs in approximately 40% of male permutation carriers and in 8% of female premutation carriers over the age of 50 [12]. Additional symptomology has since been observed, including, depression and anxiety [13], [14] and hypertension [15], [16]. Moreover, significantly increased rates of immune mediated disorders are observed in female permutation carriers but have not been observed in male permutation carriers [15], [17].

Little is known about the immune function of premutation carriers. Previous reports on immune function in female premutation carriers have shown significantly increased rates of autoimmune and autoinflammatory disorders, such as autoimmune thyroid disorders and fibromyalgia [17]. These increased rates were not observed in male carriers, but this may related to the relative rarity of these disorder in males. While not presenting with increased development of similar immune related disorders as female carriers, male carries are more prone to develop other symptomology such as FXTAS. In males with FXTAS, inflammatory profiles, similar to those seen in autoimmune and autoinflammatory disorders, have been observed suggesting that immune dysregulation exists in males as well [18]. However, it is not clear what the immune profile of premutation carriers looks like before the appearance of immune related disorders and whether this profile belies a susceptibility to autoimmune and autoinflammatory disorders.

In this current study we sought to determine whether cytokine production in healthy human female premutation carriers without evidence for preexisting immune disorders or FXTAS was altered at the cellular level. In addition, we investigated whether parallel immune findings were present in a mouse model of the fragile X premutation [19]. We hypothesize that carriers of premutation alleles will display immune profiles of dysregulation in dynamic cytokine production and altered cellular activation phenotypes.

## Methods

### Ethics Statement

The human subject portion of this study was approved by the institutional review board (IRB) at UC Davis Medical Center. All patients provided written informed consent. All experiments mice were conducted under a research protocol approved by the Institutional Care and Use Committee (IACUC) at the University of California Davis.

### Subjects

Blood samples were obtained from 36 female premutation carriers age 19–72 years [median 45.1 years (interquartile range 36.0–53.3)] who were recruited through the Fragile X Treatment and Research Center at the MIND Institute at University of California, Davis, and who participated in our genotype–phenotype study of families with fragile X between the years 2000 and 2011. Blood samples were also obtained from 15 female controls age 18–87 years [44.8 years (27.6–62.8 years)]. Subjects were excluded from the study if they had preexisting immune conditions, FXTAS, or were on immune modulating medications (methotrexate and prednisone). FXTAS was excluded utilizing criteria reported by Jacquemont et al. [20].

### Animals

Spleens were obtained from thirty-two CGG KI mice (16 males and 16 females) and thirty-two wildtype (WT) littermate mice (16 males and 16 females) used for immune studies. Mice were bred at UC Davis and were congenic on a C57BL/6J background. CGG KI mice were generated by backcrossing mice initially on a mixed FVB/N×C57BL/6J onto a C57BL/6J background over >12 generations [21]. All mice were 6 months of age at the time of experimentation to examine older adult (but not aged) immune profiles in the model. Mice were maintained by the Center for Laboratory Animal Research, at University of California, Davis and maintained at ambient room temperature on a 12 hour light/dark cycle (light on at 06∶00). Food and water were provided *ad libitum*.

### Genotyping

CGG repeat number in female premutation carriers and age matched controls were measured from dried blood spots using Southern Blot and PCR analysis as previously described [22], [23]. Genotyping of mice to verify CGG repeat length was carried out upon tail snips taken both at birth, and then again when animals were sacrificed. DNA was extracted from mouse-tail snips as previously described [24]. The number of CGG repeats were determined by PCR using the Expanded High Fidelity Plus PCR System (Roche Diagnostics, Indianapolis, IN, USA) using forward and reverse primers previously reported [24]. The DNA bands were separated using agarose gels and stained with ethidium bromide to identify their sizes.

### Cellular Isolation

Human whole blood was collected in acid-citrate-dextrose Vacutainer tubes (BD Biosciences) and upon arrival, samples are centrifuged for 2300 rpm, the plasma removed and banked at −80°C. Cells were be re-suspended in Hank’s Balanced Salt Solution, layered over 15 ml of Histopaque centrifuged at 1700 rpm for 30 minutes and peripheral blood mononuclear cells (PBMC) harvested. Isolated PBMC were resuspended in magnetic separation buffer (Miltenyi, Auburn, CA) and monocytes were isolated using CD14 microbeads (Miltenyi). The CD14 depleted cells were termed peripheral blood leukocytes (PBL) and contained mostly lymphocytes.

### Monocyte and PBL Stimulation

Monocytes and PBL’s were adjusted to a concentration of 1×10^6^ cells/ml with RPMI 1640 (Invitrogen, Carlsbad, CA) media supplemented with 10% low endotoxin, heat inactivated fetal bovine serum (Invitrogen), 100 IU/mL penicillin, and 100 IU/ml streptomycin. Monocytes were cultures in 96 well plates (200 μL) and PBLs in 12-well plates (1.0 mL). Monocytes received either media alone or lipopolysaccharide (LPS; 50 μg/mL). PBLs received media alone or PHA (20 μg/mL). Cells were incubated at 37°C, 5% CO_2_/95% air. After 24 hours, cell culture supernatants were collected and stored at −80°C until cytokine analysis.

### Murine Cell Stimulation

Mice were sacrificed and spleens collected for tissue processing. Briefly, spleens were pressed through a 100 μM nylon filter to obtain cells into suspension. Cells were then pelleted following centrifugation and red blood cells were lysed using ammonium-chloride-potassium (ACK) lysis buffer. Cells (5×10^6^/mL) were stimulated for 24 hours in a complete RPMI 1640 (Invitrogen) media supplemented with 10% low endotoxin, heat inactivated fetal bovine serum (Invitrogen), 100 IU/ml penicillin, and 100 IU/ml streptomycin (Sigma, St Louis, MO), 25 μg/ml gentimycin (Sigma), 50 μM 2-mecaptoethanol (Sigma) with media alone, LPS (1 μg/mL) or phorbol 12-myristate 13-acetate (PMA)/Ionomycin (100 ng/mL/100 uM). After 24 hours, supernatants were collect and stored at −80°C.

### Cytokine Analysis

Quantification of GM-CSF, IL-1β, IL-6, IL-10, IL-12(p40), IL-13, IL-17, IFNγ, TNFα, and MCP-1 in supernatants was carried out using murine or human multiplexing bead immunoassays (Millipore, Billerica, MA). Additionally, IL-1α was analyzed in human multiplex kits. Samples were run per manufacturer specifications. Specifically, 25 μL of supernatant was incubated with antibody-coupled beads. After a series of washes, a biotinylated detection antibody was added to the beads, and the reaction mixture was detected by the addition of streptavidin–phycoerythrin. The bead sets were analyzed using a flow-based Luminex 100 suspension array system (Bio-Plex 200; Bio-Rad Laboratories, Inc.). Unknown sample cytokine concentrations were calculated by Bio-Plex Manager software using a standard curve derived from the known reference cytokine concentrations supplied by the manufacturer. A five-parameter model was used to calculate final concentrations and values are expressed in pg/ml. The sensitivity of this assay allowed the detection of cytokine concentrations with the following limits of detection: (Human) GM-CSF (9.5 pg/mL), IL-1α (1.5 pg/mL), IL-1β (0.7 pg/mL), IL-6 (0.4 pg/mL), IL-10 (0.3 pg/mL), IL-12(p40)(10.5 pg/mL), IL-13 (0.3 pg/mL), IL-17 (0.4 pg/mL), IFNγ (0.4 pg/mL), TNFα (0.2 pg/mL), MCP-1 (1.2 pg/mL). (Murine) GM-CSF (9.5 pg/mL), IL-1β (2.7 pg/mL), IL-6 (3.4 pg/mL), IL-10 (8.0 pg/mL), IL-12(p40)(4.9 pg/mL), IL-13(10.8 pg/mL), IL-17 (0.8 pg/mL), IFNγ (2.3 pg/mL), TNFα (1.4 pg/mL), MCP-1 (9.1 pg/mL). Supernatant aliquots were free of any previous freeze/thaw cycle.

### Flow Cytometry

Following 24 hour culture with media alone or with anti-CD3 (0.5 μg/mL)/anti-CD28 (1 μl/mL) antibodies, cells were labeled with antibodies specific for cell surface markers CD3^+^, CD25^+^ and HLA-DR^+^ or isotype controls using methods previously utilized by our group [25]–[27]. In brief, cells were incubated with antibodies for 20 minutes on ice, then washed and fixed in 1% paraformaldehyde (Sigma). Multi-color flow cytometry was performed using an LSR II (BD Bioscience) equipped with a solid state Coherent Sapphire blue laser (20 mW @ 488 nm), Coherent VioFlame PLUS violet laser (25 mW @ 405 nm), and a HeNe laser (18 mW @ 633 nm). Data acquired by flow cytometry was analyzed using FlowJo Version 10 (TreeStar; Ashland, OR).

### Statistical Analysis

Data analysis was performed using STATA 12 software. Wilcoxan rank-sum tests were used to compare cytokine levels between subject groups. Spearman correlations were used to determine the association between cytokine levels and CGG repeat length. A probability value (p) of less than 0.05 was considered to be significant.

## Results

**Figure 1:**
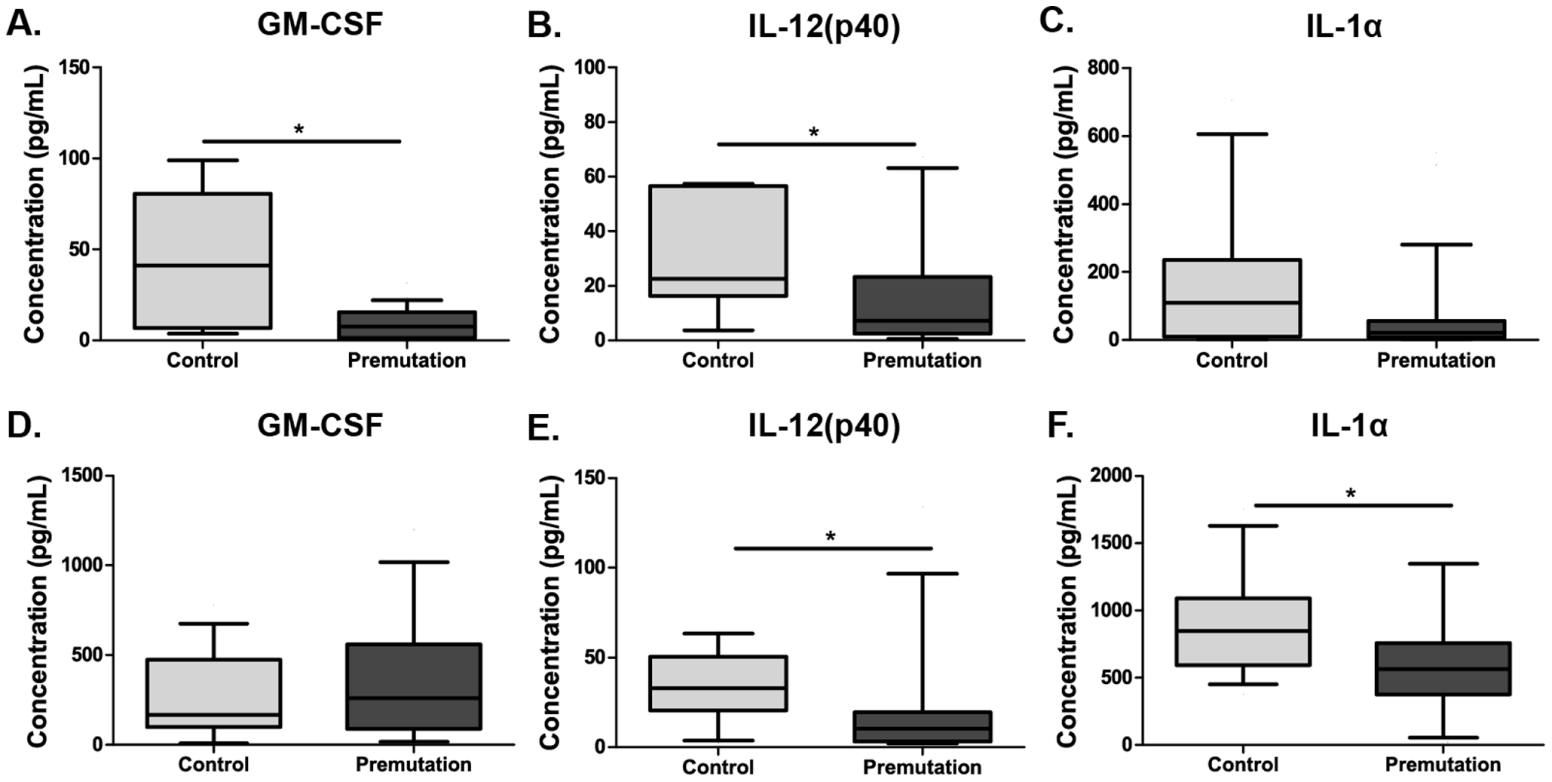
Cytokine production from human monocytes. Monocytes from premutation carriers showed significant decreased production of (**A**) GM-CSF and (**B**) IL-12(p40) but not significantly lower levels of (**C**) IL-1α when cultured with media alone. After LPS stimulation, dynamic production of both (**D**) GM-CSF and (**E**) IL-1α were significantly decreased in premutation carriers, but (**F**) IL-12(p40) levels were levels comparable between groups. *p<0.05.

Unstimulated monocytes (**Figure 1** **A–C**) from adult human female premutation carriers showed significantly decreased cytokine production compared with age-matched controls for GM-CSF [median 8.9 pg/mL (interquartile range 1.7–15.3 pg/mL) vs. 41.3 pg/mL (9.0–72.8 pg/mL); p = 0.015], and for IL-12(p40) [7.8 pg/mL (2.8–20.7 pg/mL) vs. 22.6 pg/mL (16.4–56.1 pg/mL); p = 0.042] **(** **Figure 1 **A–C**** **)**. After stimulation with LPS **(** **Figure 1 **D–F**** **)** GM-CSF, however, no longer displayed statistically significant differences between premutation carriers and controls after LPS stimulation. However, both IL-12(p40) [10.3 pg/mL (3.2–20.2 pg/mL) vs. 32.9 pg/mL (20.5–50.4 pg/mL); p = 0.029] and IL-1α [588.3.6 pg/mL (387.4–770.4 pg/mL) vs. 846.3 (607.6–1045.3 pg/mL); p = 0.040] were significantly decreased in premutation carriers.

**Figure 2:**
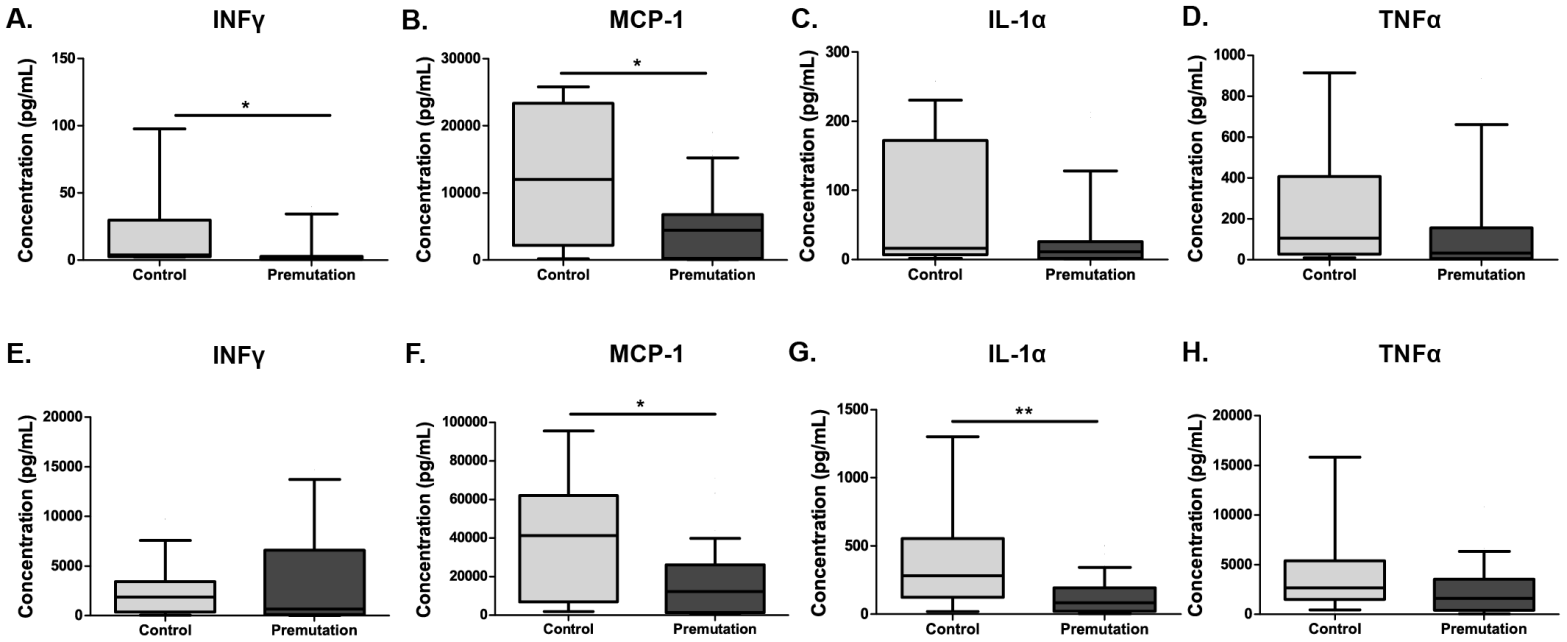
Cytokine production in human PBLs. PBLs from premutation carriers showed significantly decreased production of (**A**) IFNγ and (**B**) MCP-1; however, no apparent differences were seen in (**C**) IL-1α and (**D**) TNFα when cultured in media alone. When stimulated with a PHA, decreased production of (**E**) IFNγ in premutation carriers was seen and difference in (**F**) MCP-1 remained. Both **(G)** IL-1α and (**H**) TNFα showed trends towards decreased production, but did not reach significance. *p<0.05, and **p<0.01.

Similar to the monocytes, unstimulated PBLs **(** **Figure 2** **A–D** **)** from adult human female premutation carriers also showed decreased cytokine responses for IFNγ [1.2 pg/mL (0.7–5.1 pg/mL) vs. 4.2 pg/mL (2.4–51.0 pg/mL); p = 0.01] and for MCP-1 [4,433.3 pg/mL (33.8–6,774.8 pg/mL) vs. 12,013.8 pg/mL (2,197.0–23316.1 pg/mL); p = 0.018]. Both IL-1α (8.2 pg/mL (1.8–20.3 pg/mL) vs. 16.5 pg/mL (6.6–158.0 pg/mL); p = 0.17) and TNFα (33.3 pg/mL (6.8–144.8 pg/mL) vs. 105.1 pg/mL (27.1–408.1 pg/mL); p = 0.08) showed trends towards decreased production in premutation carriers, and approached but did not reach statistical significance. After stimulation with PHA **(** **Figure 2 E–H** **)**, IFNγ [762.0 pg/mL (122.1–4,830.0 pg/mL) vs. 2,078.2 pg/mL (386.6–3,535.0 pg/mL)] no longer showed significant differences between premutation carriers and controls, however MCP-1 from PBLs remained decreased [12,156.9 pg/mL (1,234.2–26,497.6 pg/mL) vs. 41,296.2 pg/mL (6,874.7–62,065.2 pg/mL); p = 0.021] and the production of IL-1α was decreased [84.1 pg/mL (21.7–168.1 pg/mL) vs. 282.5 pg/mL (133.7–554.0 pg/mL); p = 0.004]. TNFα [1,657.9 pg/mL (422.4–3,595.2 pg/mL) vs. 2,634.8 pg/mL (1,507.0–5,367.9 pg/mL)] displayed a trend towards decreased response, however, it did not reached statistical significance.

Decreased production of cytokines was associated with increased CGG repeat length. In unstimulated monocyte cultures from human female premutation carriers, increased CGG repeat length was associated with decreased cytokine production for GM-CSF (ρ = −0.409, p = 0.03), IL-1α (ρ = −0.360, p = 0.02), and IL-12(p40) (ρ = −0.409, p = 0.03,). After stimulation with LPS, cytokines such as IL-1β (p = 0.06), IL-12(p40) (p = 0.08) showed similar trends of decreasing levels that were associated with CGG repeat length, but failed to reach significance **(** **Table 1** **)**. Similar trends were present in the supernatants of the cultured PBLs. In media alone, production of cytokines IFNγ (r = −0.3264, p = 0.05), MCP-1 (r = −0.289, p = 0.05), and TNFα (r = −0.320, p = 0.03) were significantly associated with CGG repeat length. After stimulation with PHA, dynamic production of cytokines in PBLs show significant association with production of IL-1α (r = −0.431, p<0.01) **(** **Table 2** **)**.

**Table 1:** CGG Repeat length vs. Cytokine production from human monocytes.

	Media		LPS	
Cytokine	P	ρ	P	ρ
GM-CSF	0.03	−0.409	0.92	0.015
IL-1α	0.02	−0.360	0.27	−0.176
IL-1β	0.07	−0.274	0.06	−0.361
IL-6	0.68	−0.074	0.84	−0.049
IL-10	0.09	−0.261	0.98	0.005
IL-12(p40)	0.03	−0.409	0.08	−0.300
MCP-1	0.69	0.076	0.49	0.130
TNFα	0.06	−0.289	0.17	−0.227

Cytokine production from human monocytes were associated with CGG repeat length. Increased length of CGG showed a significantly (p<0.05) negative association with IL-1a and IL-12(p40). All p-values and spearman rho-values (ρ) were calculated by spearman test.

**Table 2:** CGG Repeat length vs. Cytokine production from human PBLs.

	Media		PHA	
Cytokine	P	ρ	P	ρ
GM-CSF	0.09	−0.260	0.53	−0.09
IFNγ	0.05	−0.326	0.33	−0.14
IL-1α	0.15	−0.238	0.00	−0.43
IL-1β	0.10	−0.245	0.28	−0.16
IL-6	0.14	−0.233	0.26	−0.17
IL10	0.16	−0.211	0.73	−0.05
IL-12(p40)	0.95	−0.010	0.50	−0.11
IL-13	0.56	0.111	0.59	0.08
IL-17a	0.65	−0.089	0.97	−0.01
MCP-1	0.05	−0.288	0.15	−0.21
TNFα	0.03	−0.320	0.08	−0.26

Cytokine production from human PBLs cultured in media alone were associated with CGG repeat length, with IFNγ, MCP-1, and TNFα all showing significantly (p<0.05) negative associations. After stimulation with PHA, however, significance with those association was diminished while a strong association (p<0.01) with IL-1a became apparent. All p-values and spearman rho-values (ρ) were calculated by spearman test.

**Figure 3:**
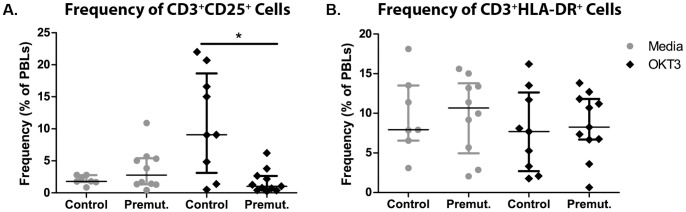
Cellular activation phenotype of PBLs. Frequencies of (**A**) CD3^+^CD25^+^ cells were significantly decreased in premutation carriers compared to age-matched controls after twenty-four out culture with anti-CD3 (OKT3) and anti-CD28. No differences were apparent in (**B**) CD3^+^HLA-DR^+^ cells between groups. *p<0.05, and p<0.01.

Phenotypic markers of activation were assessed on PBLs from premutation carriers and age-matched controls by flow cytometry. In unstimulated PBLs cultures from premutation carriers and age-matched controls similar frequencies of CD3^+^CD25^+^ and CD3^+^HLA-DR^+^ cells were observed; however, following stimulation of PBLs with anti-CD3 and anti-CD28 antibody a significant decrease was observed in the frequencies of CD3^+^CD25^+^ cells in the premutation carriers (1.8% (1.67–2.78%) vs. 2.75% (1.36–5.35%); p = 0.006). No differences in the frequency of CD3^+^HLA-DR^+^ cells **(** **Figure 3** **)** were observed between cases and controls following stimulation.

**Figure 4:**
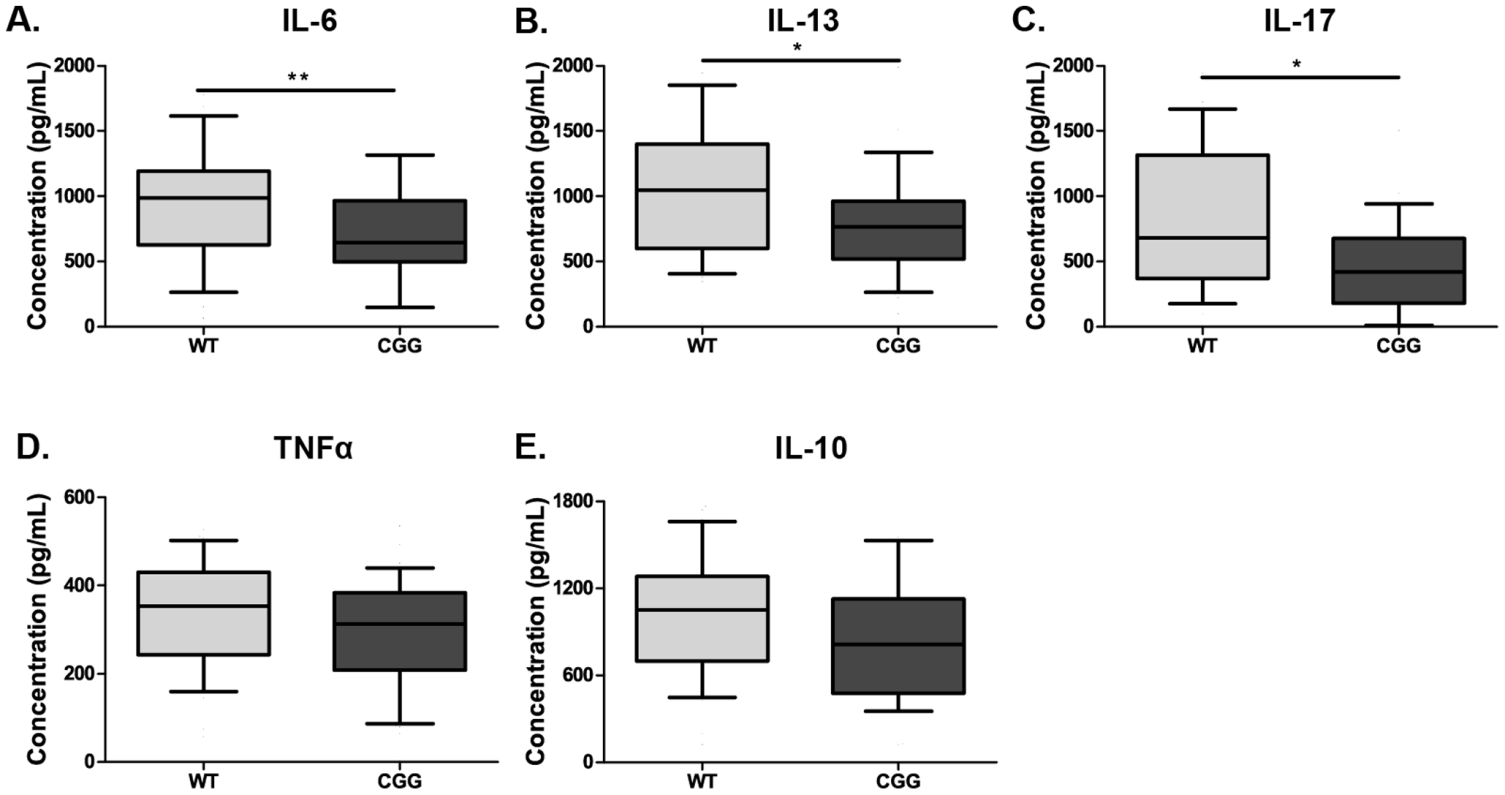
Cytokine production in mouse splenocytes. Splenocytes from CGG KI mice display significantly decreased production of (**A**) IL-6, (**B**) IL-13, and **(C)** IL-17 in responses to PMA/Ionomycin. There was a trend for decreased production of (D) TNFα, and (E) IL-10. *p<0.05, and **p<0.01.

From a potential translational viewpoint, immune responses from spleenocytes cell cultures from CGG KI mice generally showed decreased in cytokine production, although the specific cytokines differed from those seen in PBLs from human female premuation carriers. Specifically, after stimulation with PMA/Ionomycin a pronounced decrease in dynamic immune response was evident in a number of cytokines (**Figure 4**), including IL-6 [643.8 pg/mL (500.8–964.6 pg/mL) vs. 988.2 pg/mL (654.7–1,185.1 pg/mL); p = 0.008], IL-13 [766.6 pg/mL (533.9–955.3 pg/mL) vs. 1,045.3 pg/mL (606.1–1,391.9); p = 0.046], IL-17 [420.3 pg/mL (184.1–667.9 pg/mL) vs. 680.8 pg/mL (370.6–1,315.2 pg/mL); p = 0.022]. In addition, TNFα (p = 0.079) and IL-10 (p = 0.141) showed trends towards decreasing approaching but not reaching statitsical signfigance.

A negative association with cytokine production and CGG repeat length seen in PBLs in human female CGG carriers was also observed in CGG KI mice with a strong assocation between production of IL-6 and CGG repeat length (ρ = −0.330, p = 0.008).

## Discussion

Increased frequencies of autoimmune and autoinflammatory disorders have been previously reported in female premutation carriers, including a significant increased risk for developing autoimmune thyroid disorders and Fibromyalgia [15], [17]. However, little is known about the immune function of premutation carriers at a cellular level. Given the increased risk of developing autoimmune diseases and other immune related conditions, understanding the immune function of premutation carriers before onset of pathology could provide useful information both in understanding the pathophysiology associated with the premutation allele as well as to the field of autoimmunity in general. In this study we found both human and murine premutation carriers showed impaired cellular immune responses, and that human premutation carriers showed decreased relative levels of T cells expressing the cell surface marker CD25^+^, the interleukin-2 receptor alpha chain which is upregulated on T cells during cellular activation.

Autoimmune diseases are a very diverse set of disorders, each with unique pathology and associated risk factors. Both genetic studies and animal models have provided some clues as to shared factors that contribute to increased risk of developing autoimmune disorders. One theory that has arisen from this research is that a deficient rather than over active immune system may be linked to increased susceptibility for autoimmunity [28], [29]. Although not fully understood, it is believed that immunologic defects result in lack of clearance of potential pathogens, which in turn could lead to aberrant ongoing or chronic immune responses that eventually contribute to the breakdown of self-tolerance. This is evident both in some human autoimmune diseases as well as animal models of autoimmunity. In systemic lupus erythematosus (SLE), for example, individuals with deficiency in complement proteins such a C1q and C4 are associated with increased risk of developing the disorder [30]. In addition, deficiencies in antibodies, immune regulatory proteins, and cytotoxic molecules are all associated with increased risk of developing a number of systemic autoimmune disorders in both human and animals models [31]. This paradoxical association of immune deficiency and autoimmunity appears to fall on a spectrum, ranging from subclinical infection to severe infection and immunocompromise [32]. The decreased cytokine production seen in this study in both human and murine carriers of premutation alleles, as well as the previously described increase in autoimmune conditions in female premutation carriers, is consistent with the underlying theory that deficient responses may predispose for autoinflammatory conditions. This idea is further supported to some degree by anecdotal observations from our clinics which suggest that infections, stress or major surgery often pre-dates symptoms of autoimmunity in female premutation carriers (R. Hagerman personal communication).

Although our previous work had found that immune mediated disorders were only increased in female carriers, this could be for the simple reason that these disorders are much more common in females generally; meaning we lacked the power to determine the impact the premutation allele has on immune disorder development in male carriers. As male premutation carriers are less common than females, they present inherent difficulties in studying. However, to address the question of immune differences between male and females premutation carries, we looked at both males and female CGG repeat knock-in mice. Both genders showed similar immune impairments compared with controls (data not shown). This would suggest that human male carriers would also show immune impairments; while these impairment do not appear to manifest in immune related disorders, male do more commonly present with FXTAS. Recently, it was shown that males with FXTAS had inflammatory profiles similar to what is seen in many autoinflammatory disorders that are associated with female premutation carriers [18]. Although male premutation carriers do not present with the same immune disorders as female carriers it is probable that they still show similar cellular immune impairments.

In adult human female premutation carriers we saw decreased immune responses which were negatively associated with CGG repeat length**.** In monocytes isolated from premutation carriers, the most pronounced differences when compared with healthy controls were seen in production of IL-12(p40). Active IL-12 exists as a heterodimeric cytokine of 70 kDa comprising of covalently linked p40 and p35 subunit. The p40 subunit is constitutively expressed and upregulated upon activation. IL-12 drives the production of T_H_1 cytokines such as IFNγ, and is important in the immune response against viruses and intracellular bacteria. Increased levels are associated with worsening of symptoms in T_H_1 mediated autoimmune disorders such as multiple sclerosis [33]. In T_H_2 or antibody mediated disorders, such as SLE, lower levels of IL-12 have been seen in early onset SLE patients [34]. Furthermore, when IL-12 is administered to PBMCs from subjects with SLE *in vitro* it leads to decreased autoantibody production [35]. In some lupus susceptible strains of mice, IL-12 production is reduced as well [36]. Thus, potentially, the reduction in IL-12 production could explain the pattern of immune diseases seen in premutation carriers, who do not show significantly increased rates of T_H_1 mediated autoimmunity, but rather increases in antibody mediated disorders such as thyroid autoimmune disorders [17]. IL-12(p40) levels were unfortunately below the detection level in the CGG KI mouse splenocytes cultures (data not shown). Experiments looking at purified monocytes or bone-marrow derived macrophages in these animals may further elucidate the role of IL-12 in this model.

The decrease of IL-12(p40) production in the monocytes of human female premutation carriers, might contribute to the differences in the production of IFNγ observed in the PBLs, as IL-12 skews T-cells to a T_H_1 phenotype, which in turn produce IFNγ. In the CGG KI mice, IFNγ production was decreased [13,045.9 pg/mL (8,165.8–18,673.3 pg/mL) vs. 14,035.9 pg/mL (9,586.0–21,879.0)], but failed to reach significance. A lack of statistical difference may relate to the age of the mice used in these experiments. Six month old mice were chosen to better model the mean age of the human subjects (48 years of age at time of draw). However, the IFNγ production of these “aged” mice varied greatly in both CGG KI and control WT littermates. Differences may have been more apparent in younger animals. However, statistical differences were seen in the CGG KI-mice for the decreased production of inflammatory cytokines including IL-6 and IL-17. This may relate to altered T-cell function, or belie a more generalized impairment in cytokine production related to cellular function.

In PBLs of human female premutation carriers decreased production of monocyte chemoattractant protein-1 (MCP-1/CCL2) was observed with and without stimulation. MCP-1 is a chemokine that plays a major role in selectively recruiting monocytes and lymphocytes to sites of infection. It is vital for proper immune surveillance, and clearance of invading pathogens [37]. MCP-1 has both proinflammatory and anti-inflammatory roles depending on the environmental context. The proinflammatory role is in the recruitment of antigen presenting cells (APCs) and T-cells to the site of infection, the anti-inflammatory affects are dependent on the recruitment of regulatory T cells. The decreased response may indicate an inability to recruit cells at sites of infection and there after a decreased ability to clear potentially harmful pathogens from the body.

PBL’s from human female premutation carriers also had significantly decreased CD3^+^CD25^+^ cells, while the number of CD3^+^HLA-DR^+^ cells was not different **(** **Figure 3** **)**. This suggests potential differences in the activation of T-cells. CD25 is a marker of cellular activation, but within the CD25 expressing cells are a population of regulatory T cells that regulate the immune system. Further analysis on FoxP3 expression as well as functional assays to determine the differences in T effector or regulatory T cells are needed to determine if this data reflects a decrease in the ability to induce a certain type of adaptive regulatory T-cell. However, although hypothetical at this stage, deficiencies in regulatory T-cells could contribute to the increased rate of immune disorders seen in female premutation carriers.

CGG repeat length was significantly associated with decreased cytokine production in both humans and mice. In female premutation carriers, the association with CGG length and decreased cytokine production in unstimulated monocyte cultures was evident for inflammatory cytokines such a GM-CSF and IL-12(p40), and IFNγ and IL-1α. These cytokines were also negatively associated with CGG length in unstimulated and PHA stimulated PBL cultures respectively. In mice, IL-6 levels were found to negatively associate with CGG repeat length as well. The relationship with CGG repeat length and immune function may be more complicated. Mice with mid-length repeats closer to 70 CGG repeats in length appeared to be more immunologically impaired than the high-repeat length animals with repeats nearer to 150 CGG repeats, suggesting the relationship may be non-linear. This complex relationship to CGG repeat length has also been observed in human premutation carries where mid length CGG repeat size appears to have the highest rate of FXPOI [7] and psychiatric problems including depression [13], [38]. Further research is warranted to elucidate the mechanism by which increased CGG repeats contribute to immune dysregulation and the development of autoinflammatory conditions. This association between CGG repeats and immune dysfunction may have important implications for those in the general population as well. As the premutation is highly prevalent in society, nearing 1% of the female population, routine screening for CGG repeat length in the clinic may be a useful predictive tool for individuals that present with autoinflammatory conditions, although more research is warranted. In addition, targeting dysfunction based on repeat CGG’s could lead to novel therapies.

## Conclusion

In this study we report a significantly altered dynamic cytokine profile in both in human and mouse premutation carriers before development of symptomatology. The characterization of an immunological profile in CGG repeat carriers without disease may help to elucidate if an abnormal immune response may play a role in the premutation phenotype, as well as further identifying risk factors of autoimmunity in the general population, as nearly 1 in 130 women carry the premutation allele.
